# Deep sequencing and transcriptome analysis to identify genes related to biosynthesis of aristolochic acid in ***Asarum heterotropoides***

**DOI:** 10.1038/s41598-018-36316-0

**Published:** 2018-12-14

**Authors:** Xiaohan Wang, Fang Hui, Yongcheng Yang, Shihai Yang

**Affiliations:** Traditional Chinese Medicine College, Jilin Agricultural University, Changchun City, Jilin Province, 130118 China

## Abstract

*Asarum* spp. are important medicinal plants that have the potential for use in treating various types of fevers. Aristolochic acid is one of the main toxic compounds present in these plants. To improve our understanding of the biosynthetic pathway of aristolochic acid, we sequenced the transcriptome of the root and leaf tissues of *Asarum heterotropoides* and performed *de novo* sequence assembly. The data were stitched together to produce 468,357 transcripts with an N50 of 611 bp. The data were annotated with various databases (RefSeq non-redundant proteins [Nr], Swiss-Prot, Kyoto Encyclopaedia of Genes and Genomes [KEGG], Clusters of Orthologous Groups/EuKaryotic Orthologous Groups [COG/KOG], and Gene Ontology [GO]) and were annotated. There were 205,165 transcripts (43.81%) of differentially expressed genes in the roots and leaves, which were shown to be involved in biosynthesis, transport, and catabolism, and 100 genes in defence mechanisms. Three candidate transcripts (*TyrDC1*, *TyrDC2*, and *TyrDC3*) were discovered in these differential genes. *TyrDC* may be a key enzyme in the biosynthesis pathway of aristolochic acid identified using quantitative reverse transcription-polymerase chain reaction (qRT-PCR) and high-performance liquid chromatography (HPLC). The transcriptome data and analysis presented here lay the foundation for further research into these important medicinal plants.

## Introduction

*Asarum heterotropoides* Fr. Schmidt var. *mandshuricum* (Maxim.) Kitag. is a medicinal plant that dispels chills associated with exposure of the body to cold produced *in vivo*, and it has analgesic and antitussive effects. It is an important medicine for treating wind chill, headache, and cough. Liu *et al*.^1^ and Zeng *et al*.^2^ identified various components from the volatile oils of *Asarum heterotropoides* Fr. Schmidt var. *mandshuricum* (Maxim.) Kitag. and *Asarum siedboldii* Miq. var. *seoulense* Nakai., including methyleugenol, safrole, elemicin, and demethylcoclaurine. L-sesamin and aristolochic acids A, B, C, and D were identified in non-volatile oils^3^. The current edition of the Chinese Pharmacopoeia^4^ lists medicinal *Asarum*-based plant materials as the dried roots and rhizomes of *A. heterotropoides* Fr. Schmidt var. *mandshuricum* (Maxim.) Kitag., *A. siedboldii* Miq. var. *seoulense* Nakai., and *A. siedboldii* Miq. *Asarum* has a wide geographical distribution; *A. heterotropoides* Fr. Schmidt var. *mandshuricum* (Maxim.) Kitag., and *A. siedboldii* Miq. var. *seoulense* Nakai. grow as undergrowth or on mountainous wetland^4^. The main producing areas are Jilin, Liaoning, and Heilongjiang in China. *A. siedboldii* Miq. grows in damp places near creeks or rocky areas of Shanxi, Gansu, Zhejiang and other places in China^5^.

Many Chinese herbal medicine formulations containing aristolochic acid I are banned or restricted^6–8^. To effectively use *A. heterotropoides* and other aristolochic acid-containing medicinal herbs, various traditional Chinese medicine formulation have been studied, mostly with a focus on reducing their toxicity by preparing slices from the medicinal plant parts. Traditional breeding can also remove plant toxicity. The traditional Chinese medicine involves the use of concoctions (Paozhi) and solvents or diluents such as wine, vinegar, salt, and honey are used to treat slices of traditional Chinese medicine formulations. Only slices of traditional Chinese medicine preparations can be used to treat diseases. Traditional Chinese medicine methods such as concocting and cooking show reasonable compatibility and can remove aristolochic acid, but completely eliminating its toxicity is difficult. Genetically removing or reducing the aristolochic acid content in *A. heterotropoides* is the most effective method. Although this has not been previously performed, it is possible to elucidate the synthesis pathway of aristolochic acid in plants. Therefore, in this study, we identified genes with a strong correlation in the synthesis pathway of aristolochic acid using high-throughput sequencing and transcriptome data analysis. Reduction or blockade of the gene expression using gene silencing could reduce the toxicity of aristolochic acid-containing plants to facilitate their safe use and breeding of new plant varieties with high efficacy and low toxicity.

Among the plants of *Aristolochiaceae*, there are reports in the literature that *Aristolochia L*. plants *Aristolochia fimbriata* and *Aristolochia tagala* have available expressed sequence tags (ESTs) and transcriptome data, respectively^9,10^, while *Saruma henryi* Oliv. has transcriptome information^11^. The chloroplast genome of *A. heterotropoides* was sequenced in *Asarum L*. in 2017^12^. Presently, there is no literature report of the sequencing of whole genomes or transcriptomes of *A. heterotropoides*. In this experiment, the roots and leaves of *A. heterotropoides* were collected on 3 September, 2016, and a cDNA library was established. Transcriptome data were obtained using high-throughput sequencing. An analysis of the transcriptome data determined that the tyrosine decarboxylase (*TyrDC*) family of enzymes is involved in the biosynthesis pathway of aristolochic acid in *A. heterotropoides*, and the full-length gene sequences of three enzymes were identified.

## Results and Discussion

### Illumina sequencing and read assembly

**Figure 1:**
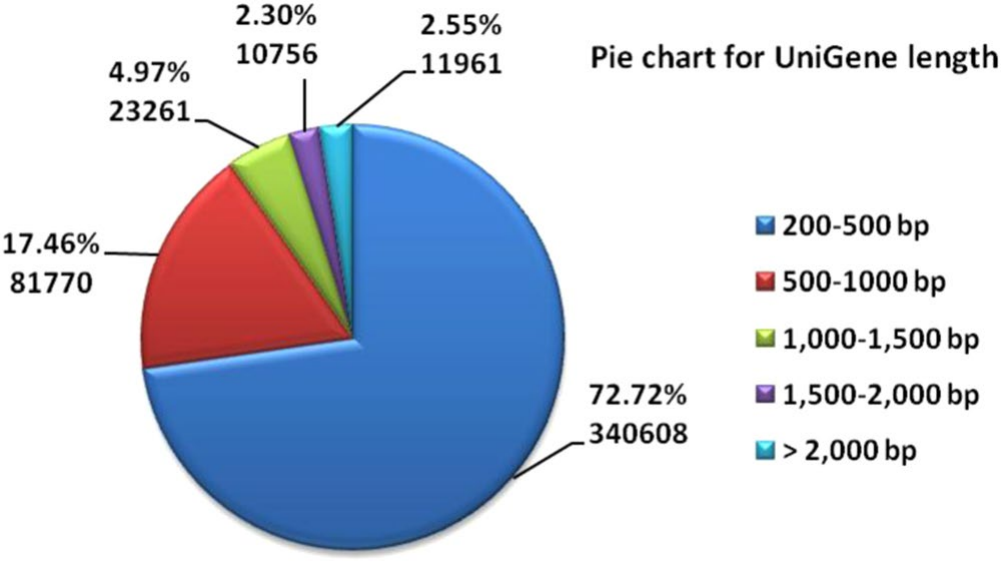
Overview of transcriptome assembly data showing the size distribution of transcripts.

The transcriptome data for *A. heterotropoides* were obtained, and 268,492,920 raw readings were generated with a read length of 100 bp. After trimming to remove adaptors, primer sequences, polyA tails, and short and low-mass sequences, 265,808,030 (99.0%) high-quality reads were recovered (Table 1). Trinity assembly data were used to assemble sample data from the beginning, and the assembly result was passed through the sequence. Clustering was used for further sequence splicing and de-redundancy processing to obtain long non-redundant UniGene sequences. After data processing, 468,357 UniGene sequences were included, and the N50 was 611 bp. The UniGene sequence had a size range of 201–15,756 bp and an average size of 507.36 bp. In the 200–500, 500–1,000, 1,000–1,500, 1,500–2,000, and >2,000 bp size ranges there were 340,608 (72.72%), 81,770 (17.46%), 23,261 (4.97%), and 10,756 (2.30%), and 11,961 (2.55%), respectively, as shown in Fig. 1.

**Table 1:** Sequencing raw Data and Filtered data quality statistics.

Sample	length	Reads	Bases	Q20 (%)	Q30 (%)	GC (%)	N (ppm)
**Sequencing raw Data quality statistics**							
Leaf-1	150	44162550	6624382500	95.45	89.71	43.44	630.62
Leaf-2	150	43709506	6556425900	96.29	91.41	44.39	718.62
Leaf-3	150	44445702	6666855300	95.69	90.29	43.25	637.75
Root-1	150	50545156	7581773400	96.52	91.81	45.28	773.45
Root-2	150	41393484	6209022600	96.24	91.24	45.28	768.23
Root-3	150	44836522	6725478300	95.69	89.99	45.18	634.39
**Filtered data quality statistics**							
Leaf-1	147.35	43557996	6418359982	96.35	90.87	43.46	16.68
Leaf-2	147.5	43192306	6370737693	97.06	92.38	44.42	12.15
Leaf-3	147.11	43795384	6442723147	96.64	91.48	43.24	16.42
Root-1	147.93	50018222	7398987367	97.18	92.63	45.31	8.03
Root-2	147.94	40926052	6054787512	96.98	92.15	45.3	7.74
Root-3	147.91	44318070	6555110250	96.43	90.94	45.2	16.11

### Functional annotation

Functional annotation of assembled transcripts provides an insight into their molecular functions and biological processes in an organism. We compared the transcript data with the Gene Ontology (GO) database and used the Basic Local Alignment Search Tool (BLAST) to search data in several public databases.

**Figure 2:**
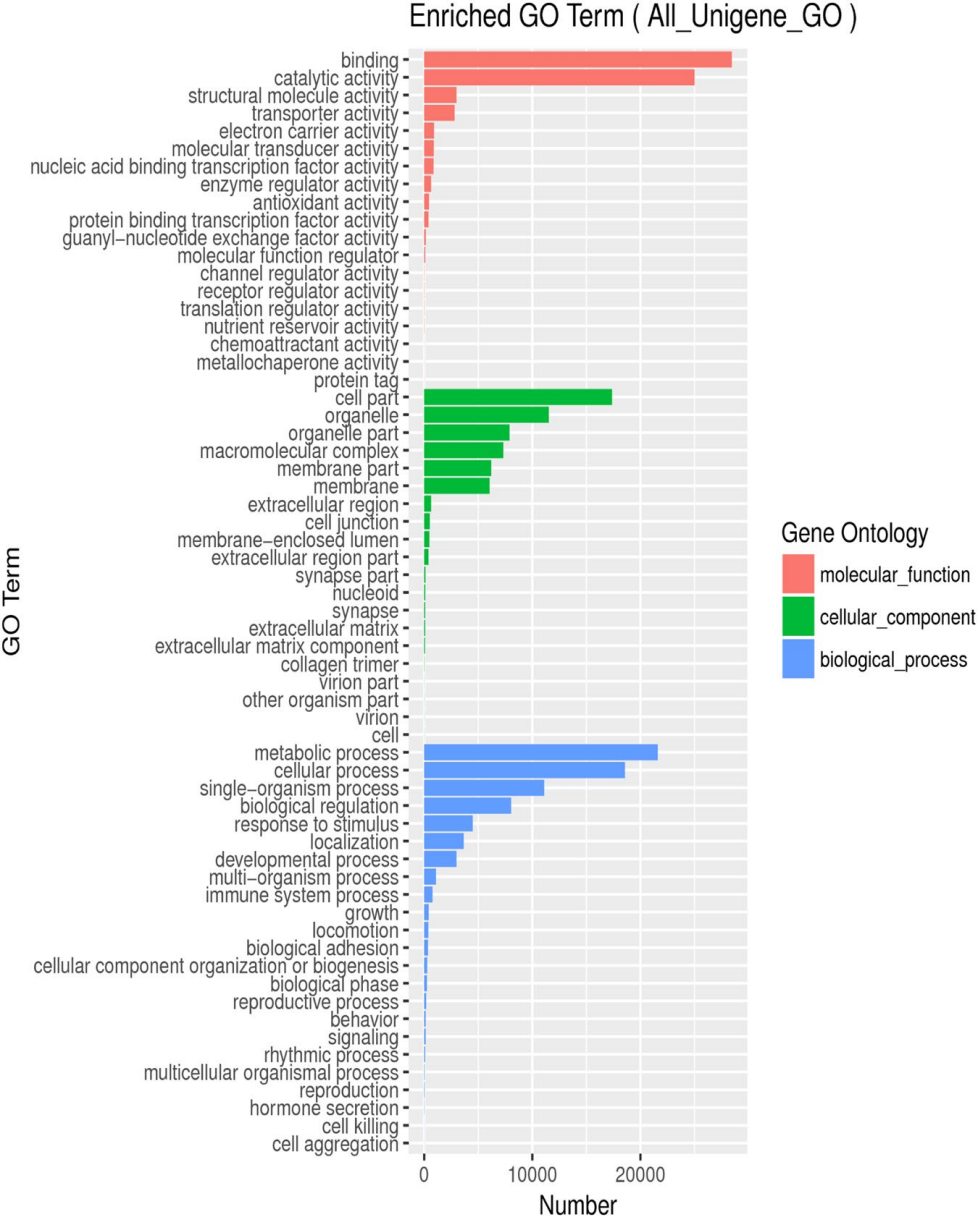
Gene numbers of frequencies and mean of transcripts matching GO terms.

GO is a biological information network initiative with a three-tier system that defines and describes the function of genes and proteins as well as other gene products. The GO database consists of three major categories: molecular functions, cell components, and biological processes. Based on the differential transcript annotations to GO, we focused on the three categories that matched the molecular functional domain the most: “binding”, “catalytic activity”, and “structural molecule activity” (approximately 28,000, 25,000, and 2,600, respectively). The three most abundant categories under cellular components were “cell part”, “organelle”, and “organelle part” (approximately 17,000, 11,500, and 7,500, respectively). The three most common categories of biological processes were “metabolic process”, “cellular process”, and “single-organism process” (approximately 22,000, 18,500, and 11,000, respectively). After comparison and analysis, we mapped the GO functional classification annotation (Fig. 2).

**Figure 3:**
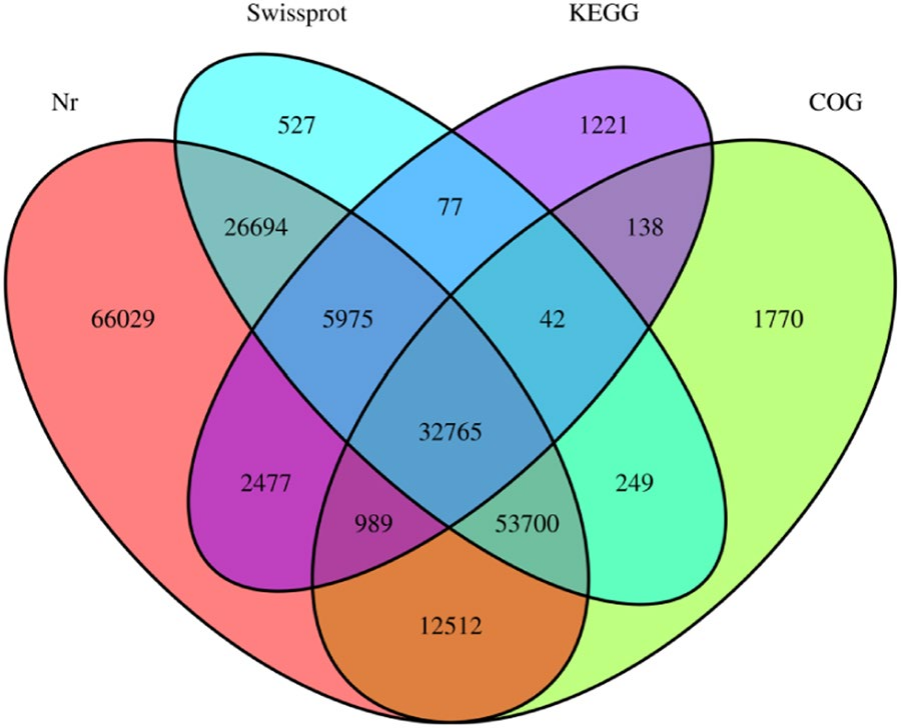
Venn diagram representing the number of DETs among *A. heterotropoides*.

The transcriptome data of *A. heterotropoides* were tagged using the BLAST search data in the public database (Table 2) and Venn diagrams of the UniGene database annotation results were constructed (Fig. 3).

**Table 2:** UniGene database commentary statistics.

Total_UniGenes	Total_annotated_UniGenes	Nr	COG	Swissprot	KEGG
468357	205165	201141	102165	120029	43684

Of the 468,357 transcripts, 205,165 (43.81%) UniGene sequences were annotated. They consisted of 201,141 (42.95%), 120,029 (25.63%), 102,165 (21.81%), and 43,684 (9.33%) in Nr, Swiss-Prot, COG, and Kyoto Encyclopedia of Genes and Genomes (KEGG) in the total UniGenes list, respectively. The remaining 56.19% of transcripts were not annotated, indicating that there was no significant protein match in the database. Furthermore, there were unreported or multifunctional proteins in the *A. heterotropoides* transcript or genes derived from conserved untranslated regions and long-chain non-coding RNA.

**Figure 4:**
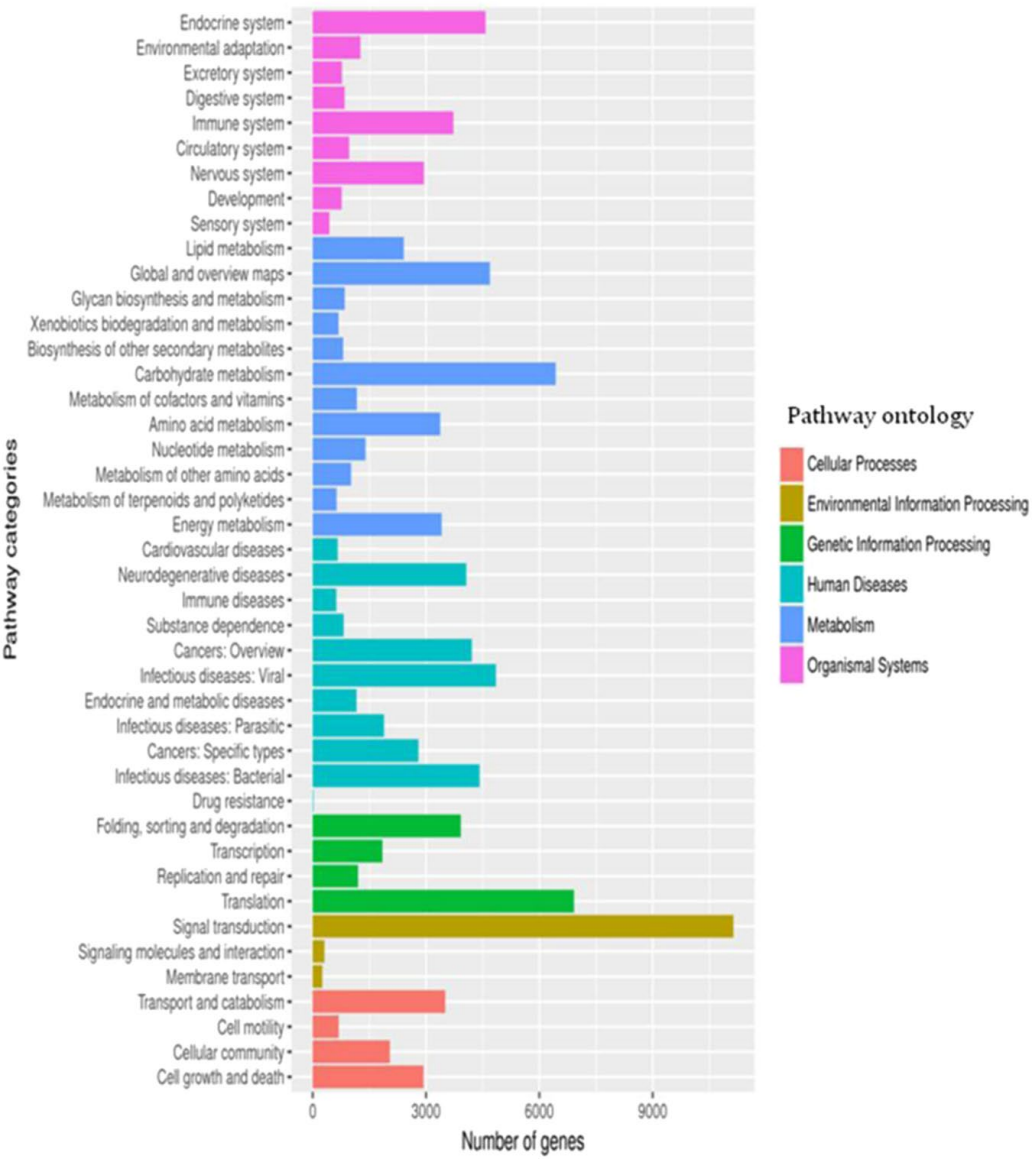
KEGG pathway classifcation map. Genes were divided into fve branches according to the biological pathways they participated in: (Red), Cellular Processes; (Yellow), Environmental Information Processing; (Green), Genetic Information Processing; (Blue), Human Diseases; (Deep Blue), Metabolism; (purple), Organismal Systems.

The KEGG database simulates biological reactions through computational analysis and calculates and predicts metabolic pathways and functions of cellular gene products. It can provide integrated metabolic pathways and relationships between pathways, and comprehensively annotate enzymes that catalyse each step of the reaction. From this analysis, we found that 43,684 transcripts could be assigned to 311 pathways in six major categories. The six main categories were “organismal systems” “metabolism,” “genetic information processing”, “human diseases”, “environmental information processing”, and “cellular process”. The highest numbers of UniGene sequence transcripts included in each category were as follows: “endocrine system”, 4,575 (10.47%); “carbohydrate metabolism”, 6,433 (14.73%); “translation”, 6,915 (15.83%); “infectious diseases: viral”, 4,848 (11.10%); “signal transduction”, 11,136 (25.49%); and “transport and catabolism”, 3,510 (8.03%), as shown in Fig. 4. In addition, we also noted some enzymes that were enriched in the KEGG pathway for common secondary metabolites (Table 3). These included three KEGG pathways associated with alkaloid synthesis, which included 200 UniGene sequences. There were two KEGG pathways associated with tyrosine anabolism containing 431 UniGene sequences, which may be related to the aristolochic acid synthesis pathway.

**Table 3:** Secondary metabolism pathways in *A. heterotropoides*.

Pathway	Gene_num
ko00950 Isoquinoline alkaloid biosynthesis	97
ko00901 Indole alkaloid biosynthesis	3
ko00400 Phenylalanine, tyrosine and tryptophan biosynthesis	209
ko00350 Tyrosine metabolism	222
ko00960 Tropane, piperidine and pyridine alkaloid biosynthesis	100
ko00940 Phenylpropanoid biosynthesis	286
ko00900 Terpenoid backbone biosynthesis	253
ko00902 Monoterpenoid biosynthesis	10
ko00904 Diterpenoid biosynthesis	28
ko00909 Sesquiterpenoid and triterpenoid biosynthesis	36
ko00944 Flavone and flavonol biosynthesis	2
ko00941 Flavonoid biosynthesis	51
ko00130 Ubiquinone and other terpenoid-quinone biosynthesis	144
ko00942 Anthocyanin biosynthesis	3
ko00860 Porphyrin and chlorophyll metabolism	217

**Figure 5:**
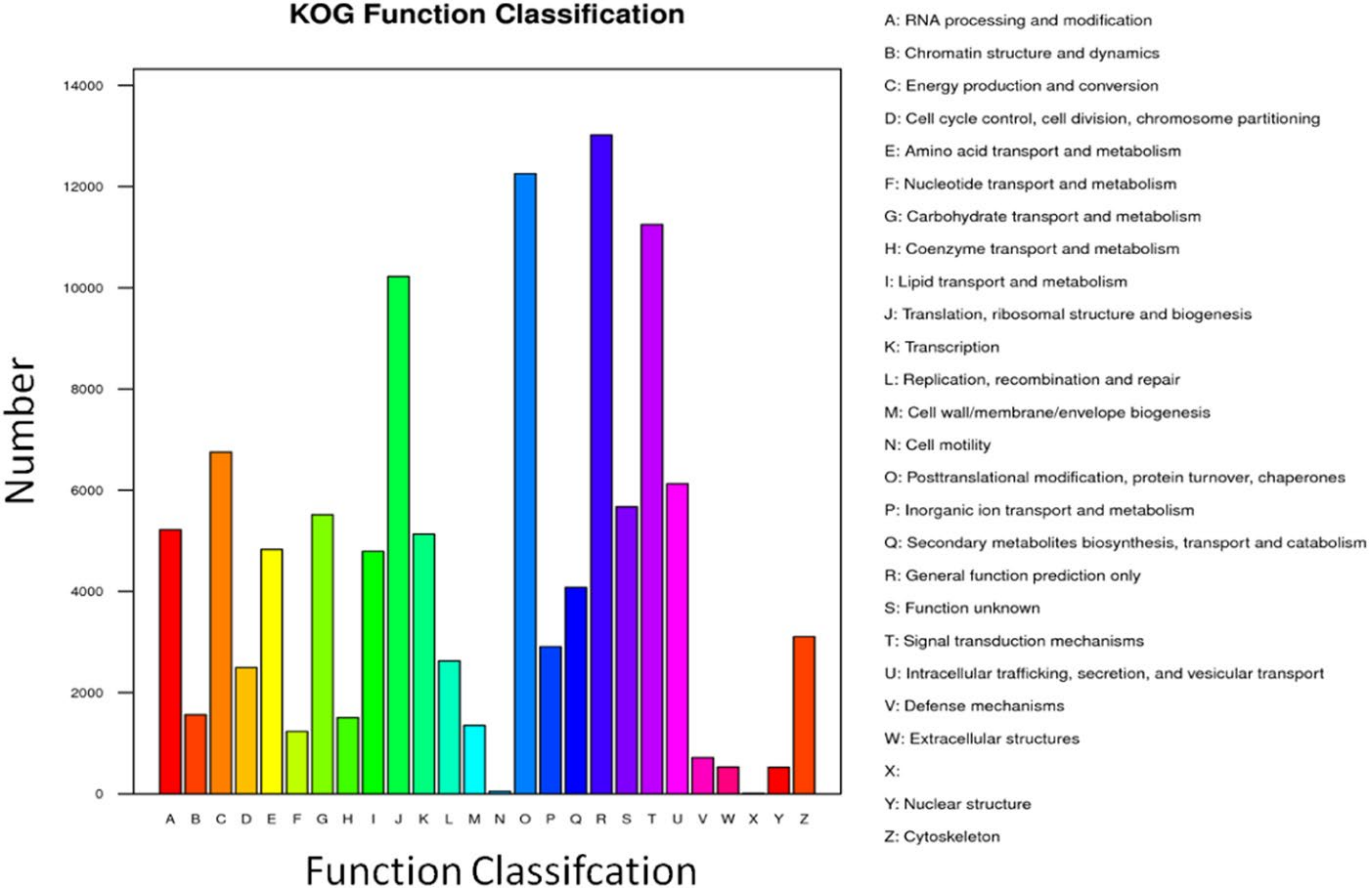
KOG Function classifcation map.

The COG/EuKaryotic Orthologous Groups (KOG) database is based on the phylogenetic relationship of encoded proteins of the complete genomes of bacteria, algae, and eukaryotes. The alignment could annotate a specific protein sequence to a specific COG, and each cluster of the COG is composed of orthologous sequences, which enables the identification of sequence functions. This analysis identified 102,165 UniGene sequences that were annotated (Fig. 5). The most common was the general function prediction, which consisted of 13,020 sequences, accounting for 12.74% of the total annotation. The second and third were adopted from the UniProtKB entry post-translational modification, protein turnover, chaperones, and signal transduction mechanisms, and they had 12,254 and 11,251 UniGene sequences, accounting for 11.99% and 11.01%, respectively of the total annotation ratio. Among these notes, we were more concerned with amino acid transport and metabolism (4,831 and 4.73%), secondary metabolite biosynthesis, transport, and catabolism (4,080 and 3.99%), and defence mechanisms (715 and 0.70%).

### Differential gene expression analysis

**Figure 6:**
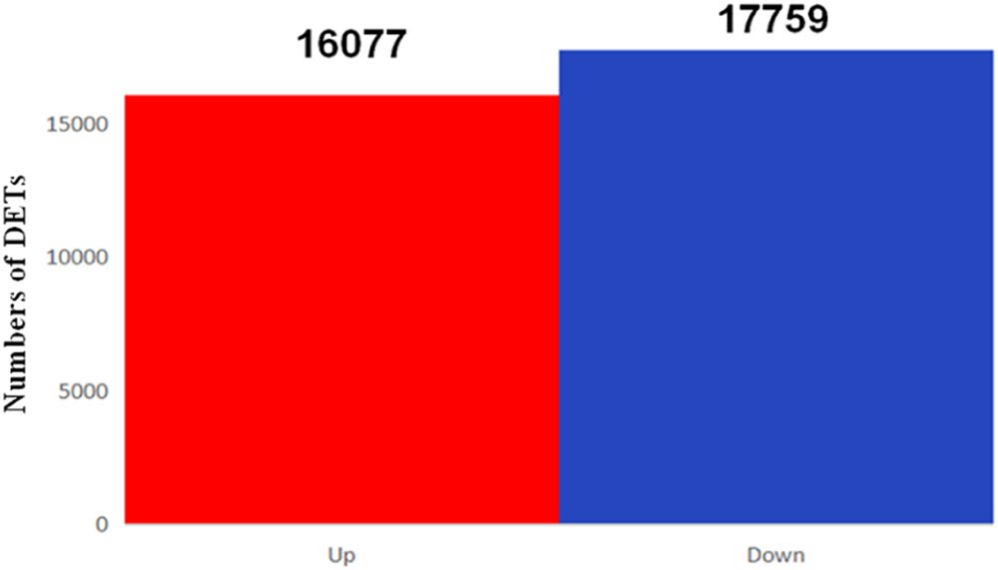
Significantly Differentially Expressed Transcripts between Leaves and Roots in *A. heterotropoides*.

**Figure 7:**
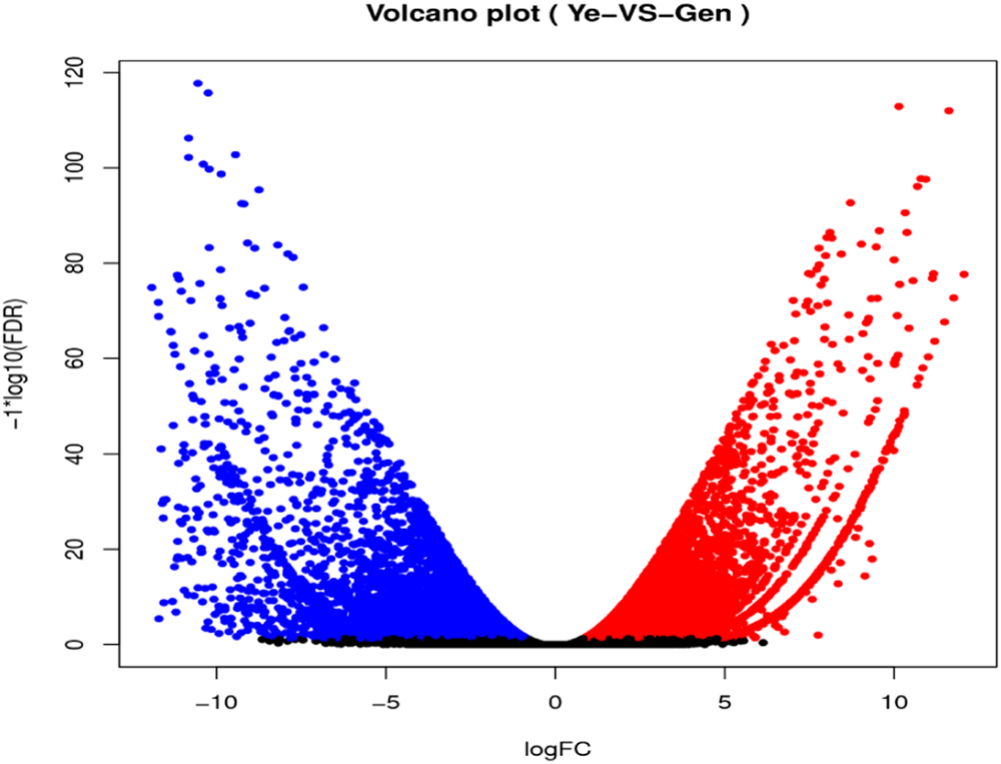
Volcano of Significantly Differentially Expressed Transcripts between Leaf and Root in *A. heterotropoides*.

To study differential gene expression in different tissues, we used bowtie2 (2.1.0) to map high-quality reads from individual samples onto the *A. heterotropoides* transcriptome. Approximately 67.97–76.14% of the total reads were successfully located in the transcriptome (Table 4). Differentially expressed transcripts were screened for more than 2-fold changes in differential gene expression and false discovery rate (FDR) ≤0.05. Substantial transcriptional differences were observed in pairwise comparisons between different tissues. We used the *A. heterotropoides* UniGene of leaves as the control compared to UniGene of roots, and a comparison of the data for the leaves and roots showed that 16,077, 17,759, and 33,836 genes were up-regulated, down-regulated, and differentially expressed, respectively (Figs 6 and 7).

**Table 4:** Comparison Table.

Samples	Total_reads	Total_mapped	Unique_mapped	Multi_mapped	Mapped (%)	Unique (%)
Leaf -1	43557996	30994358	24074268	6920090	71.16	55.27
Leaf -2	43192306	31780440	24724786	7055654	73.58	57.24
Leaf -3	43795384	29768932	23201770	6567162	67.97	52.98
Root -1	50018222	38084954	30253176	7831778	76.14	60.48
Root -2	40926052	30801496	24492586	6308910	75.26	59.85
Root -3	44318070	33477706	26489654	6988052	75.54	59.77

**Figure 8:**
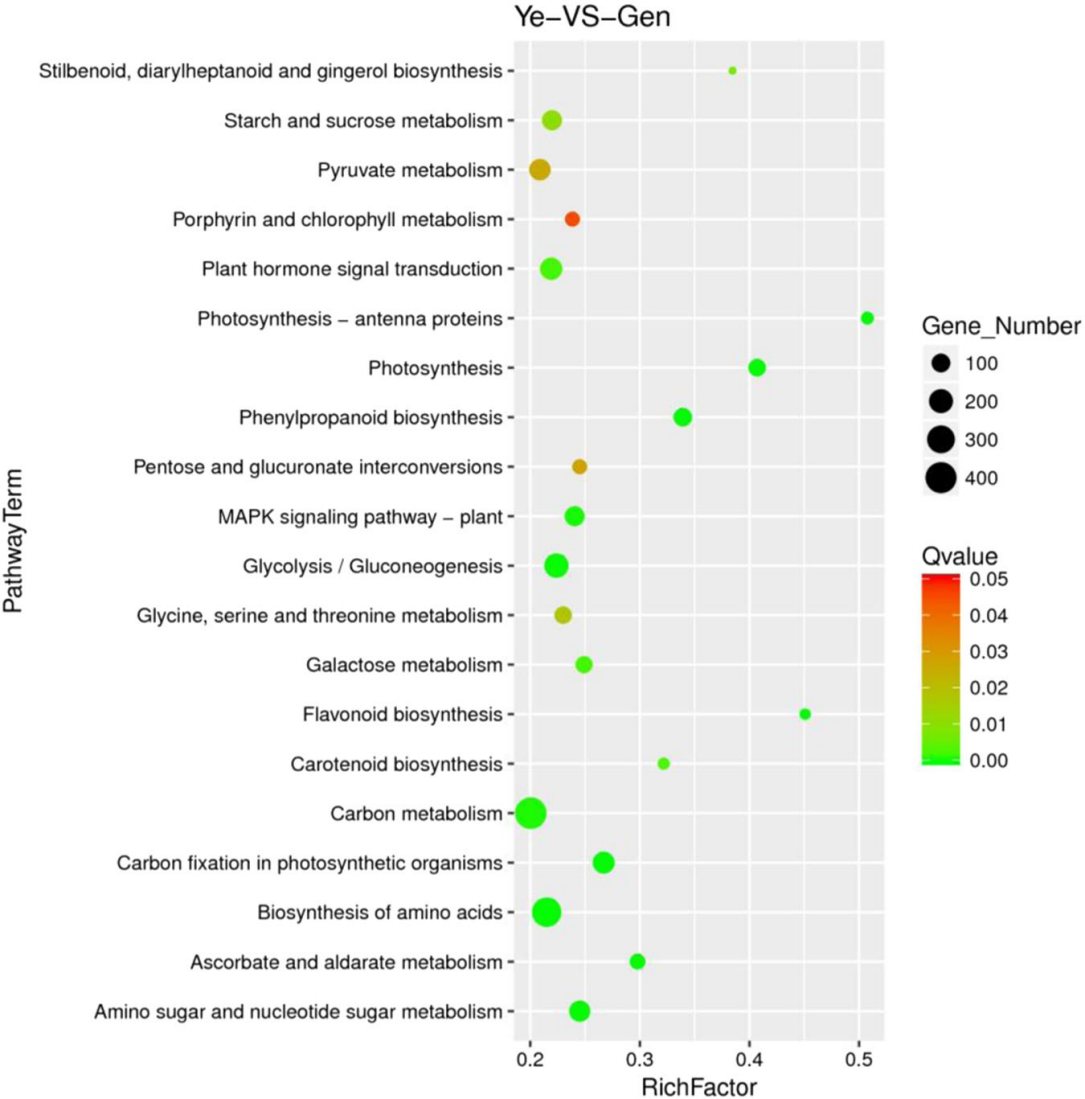
The KEGG enrichment scatter plot.

The KEGG enrichment scatter plot is a graphical representation of the KEGG enrichment analysis results and as shown in Fig. 8, the degree of KEGG enrichment was measured using the Richfactor, Q-value, and the number of genes enriched in this plant metabolic network. The Richfactor refers to the ratio of the number of differentially expressed to the total number of annotated genes in the plant metabolic network entry. The larger the Richfactor value, the greater the degree of enrichment. The Q-value, which is the P-value after multi-hypothesis test correction has a range of 0–0.05, and the closer the value is to zero, the more significant the enrichment. Based on an analysis of this graph, we concluded that carbon metabolism accounted for the largest proportion of enriched genes (365, 7.33%), followed by biosynthesis of amino acids (345, 6.92%), starch and sucrose metabolism (173, 3.47%), plant hormone signal transduction (165, 3.31%), phenylalanine metabolism (83, 1.67%), phenylpropanoid biosynthesis (95, 1.91%), and flavonoid biosynthesis (23, 0.46%). These plant metabolic networks are related to the secondary metabolic plant metabolic networks.

**Figure 9:**
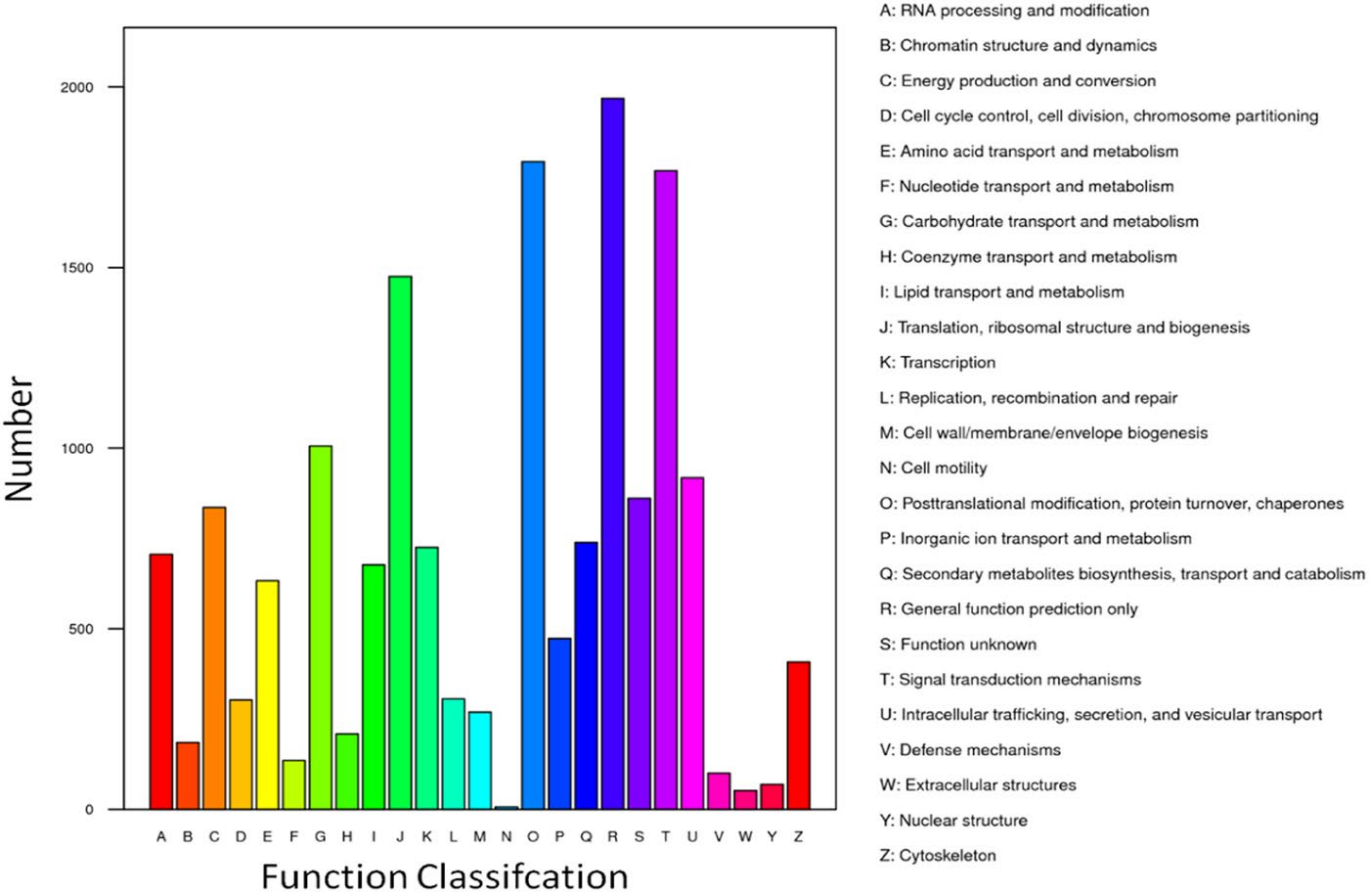
KOG Function classifcation map in differentially expressed genes.

In the COG/KOG annotation analysis of differentially expressed genes, we focused on the biosynthesis, transport, and catabolism of secondary metabolites (739); amino acid transport and metabolism (633); and defence mechanisms (100) as shown in Fig. 9.

### Putative genes involved in the aristolochic acid I biosynthesis plant metabolic network

**Figure 10:**
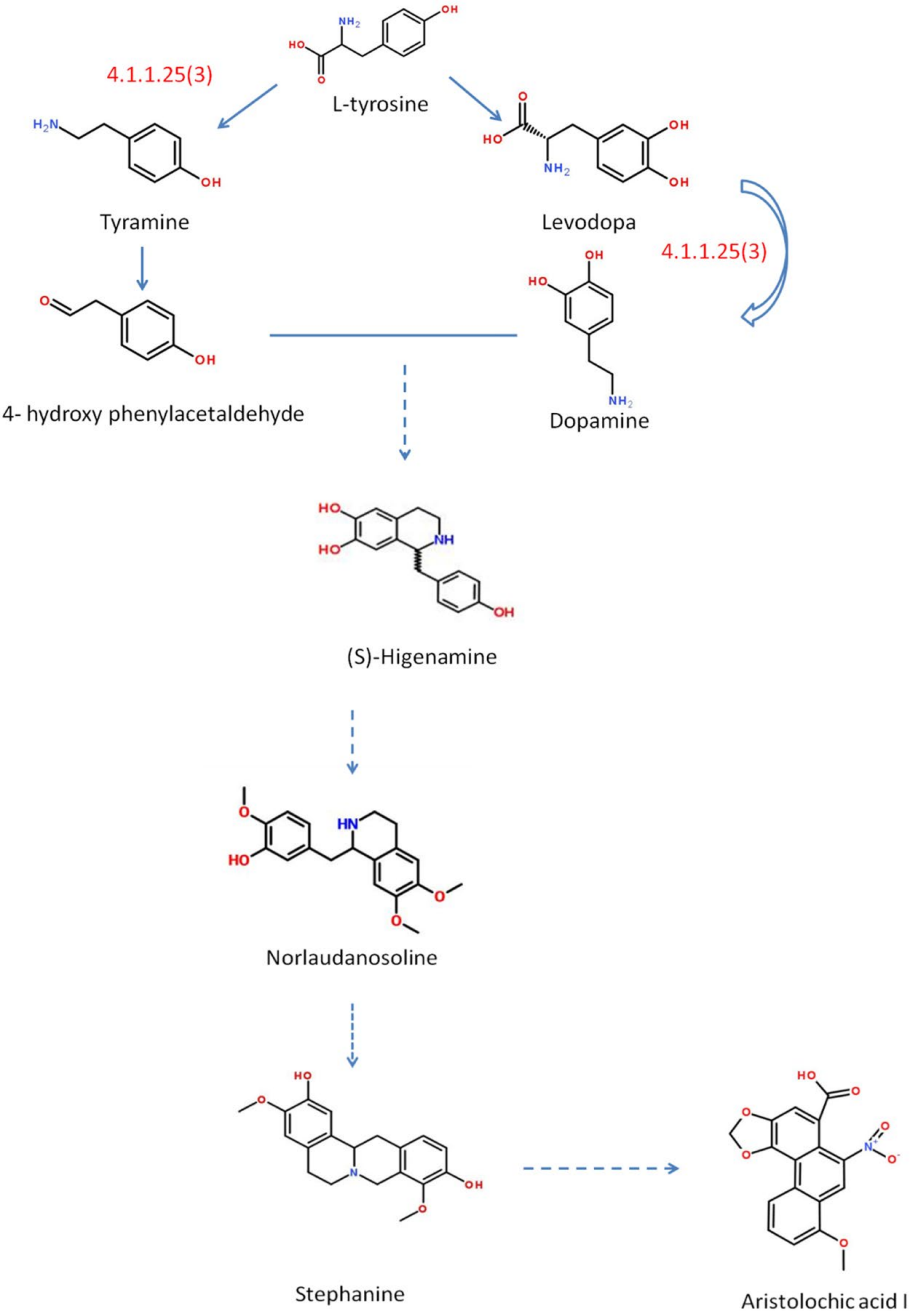
Proposed plant metabolic network of aristolochic acidIbiosynthesis. 4.1.1.25 represents tyrosine decarboxylase. Adapted from the literature published by Comer F (1969)^14^.

Comer *et al*.^13^ reported the formation of radioactive aristolochic acid I when [β-14C,15N]-tyrosine was added to *Aristolochia sipho*. The synthesis pathway of aristolochic acid I is speculated to be tyrosine → tyramine → 4-hydroxy-phenyl-acetaldehyde → (s)-nor-amethoxine → (S)-Nor Laudanine → orientaliue → stephanine → aristolochic acid I, adapted from the literature published by Comer (1969)^14^ (Fig. 10). Schutte *et al*.^14^ applied DL-[4-^14^C]-allo-labardine hydrochloride and DL-[4-^14^C]-tetrahydropapamine hydrochloride to the stem of *A. sipho*, and radioactive aristolochic acid I was only obtained where allotropone was smeared, but at a low concentration. Therefore, stephanine has not been proven to be an intermediate product of aristolochic acidI biosynthesis.

Combining the results of the analysis of the putative aristolochic acid synthesis plant metabolic network and transcriptome data, it was determined that possible precursor compounds for the synthesis of aristolochic acid were in KEGG00950 and 00350. Differentially expressed genes from these plant metabolic networks in the transcriptome data were screened, and 34 genes with strong correlation were obtained (Supplemental Tables 1 and 2). Among these candidate genes, there were 30 enzymes prior to tyrosine decarboxylase and 4 tyrosine decarboxylases. We first analysed and cloned two predicted enzymes that were enriched near stephanine, but the cloning was unsuccessful because the size of the predicted gene fragment was too small (approximately 200 bp) or the copy number of the gene itself was low. *A. heterotropoides* has a high content of active ingredients and a small amount of aristolochic acid I in the three basic plant species, as stipulated by the Chinese Pharmacopoeia, which may be why it is difficult to enrich the terminal enzyme gene in the entire plant metabolic network in transcripts.

Aristolochic acid is the first phenanthrene-containing acid found in nature and its biosynthetic pathway involves multiple enzyme gene. The details of the plant metabolic network remain unclear. Tyrosine deaminase is involved in nitrogen metabolism and important nodes generated by nitrogen compounds. Therefore, we chose to study its correlation with aristolochic acid synthesis. In previous studies, we preliminarily determined that there may be a correlation between *TyrDC* enzyme activity and aristolochic acid Icontent using various experiments, and speculated that *TyrDC* might be related to the biosynthesis pathway of aristolochic acidI.

Based on the above analysis, we chose to further study the *TyrDC* gene.

### Bioactive compound biosynthesis and accumulation

To detect possible associations between *TyrDC* gene expression and metabolite production, we determined the content of aristolochic acidI in roots and leaves (Supplemental Fig. 1). The aristolochic acid I content in the roots was higher than that in the fresh leaves, whereas it was higher in the leaves after drying than it was in the roots (Table 5). We also included the content of aristolochic acid I in the stems and flowers as a reference^15^ (Table 6). However, since the stems and flowers are not used as medicine, we did not use them as samples for transcriptome sequencing. Aristolochic acid I is toxic to humans and other species and, therefore, its high content could be a defensive mechanism of plants against predators. The expression profile of metabolite biosynthetic genes in different tissues correlated with the metabolite content.

**Table 5:** Aristolochic acid I content in roots and leaves of *A. heterotropoides*.

	Weight	Root μg·g^−1^	Leaf μg·g^−1^
Aristolochic acidI	Fresh weight	89.4955 ± 2.4526	46.1213 ± 1.4298
	Dry weight	122.5328 ± 4.3668	1939.3629 ± 9.2510

**Table 6:** Aristolochic acid I content in stems and flowers of *A. heterotropoides*.

	Stem μg·g^−1^	Flower μg·g^−1^
Aristolochic acid I	1710.6289 ± 7.5628	3243.1121 ± 8.0294

### Quantitative reverse transcription-polymerase chain reaction (qRT-PCR) validation of different expression patterns

**Figure 11:**
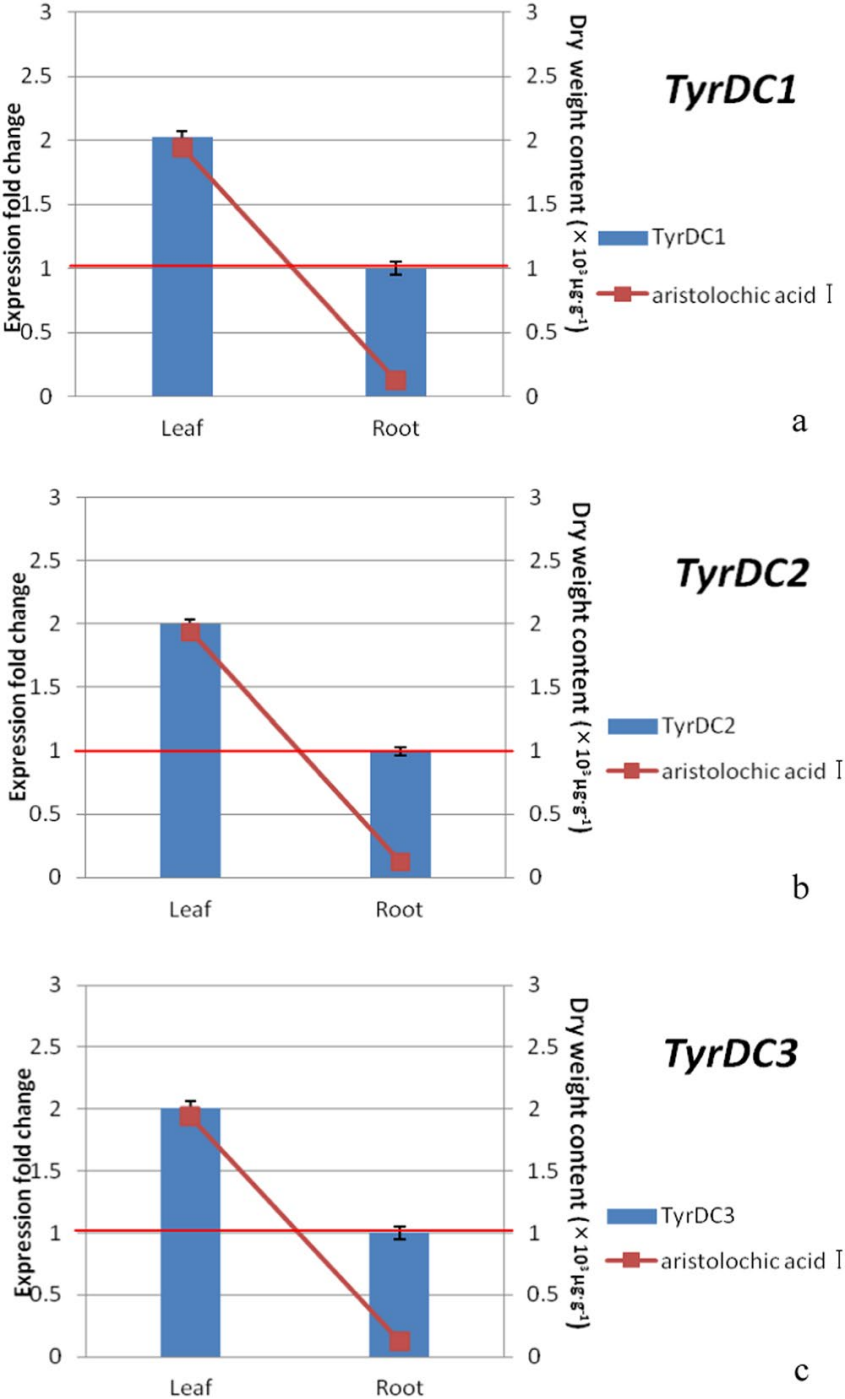
The expression pattern of *TyrDCs* across different tissues. a, b, and c represent the content of aristolochic acidIand the relative expression levels of *TyrDC1*, *TyrDC2*, and *TyrDC3* in *A. heterotropoides*, respectively. The expression levels of *TyrDCs* in leaves relative to the values of roots (control), which were given an arbitrary value of 1.

To validate the transcriptome analysis data and better understand the molecular basis of the metabolic plant metabolic networks involved in aristolochic acid biosynthesis, we selected three transcripts of *TyrDCs* and determined their expression patterns using quantitative reverse transcription-polymerase chain reaction (qRT-PCR). The results of the qRT-PCR in the transcriptome data were compared to verify that the transcriptome results were authentic and reliable. Based on the analysis of the expression of tyrosine decarboxylase in *A. heterotropoides* and leaves and the content of aristolochic acid I, the results confirmed that tyrosine decarboxylase had a strong positive relationship with the biosynthesis of aristolochic acid I (Fig. 11 and Supplemental Table 3).

### Full-length clones of *TyrDC* family

**Figure 12:**
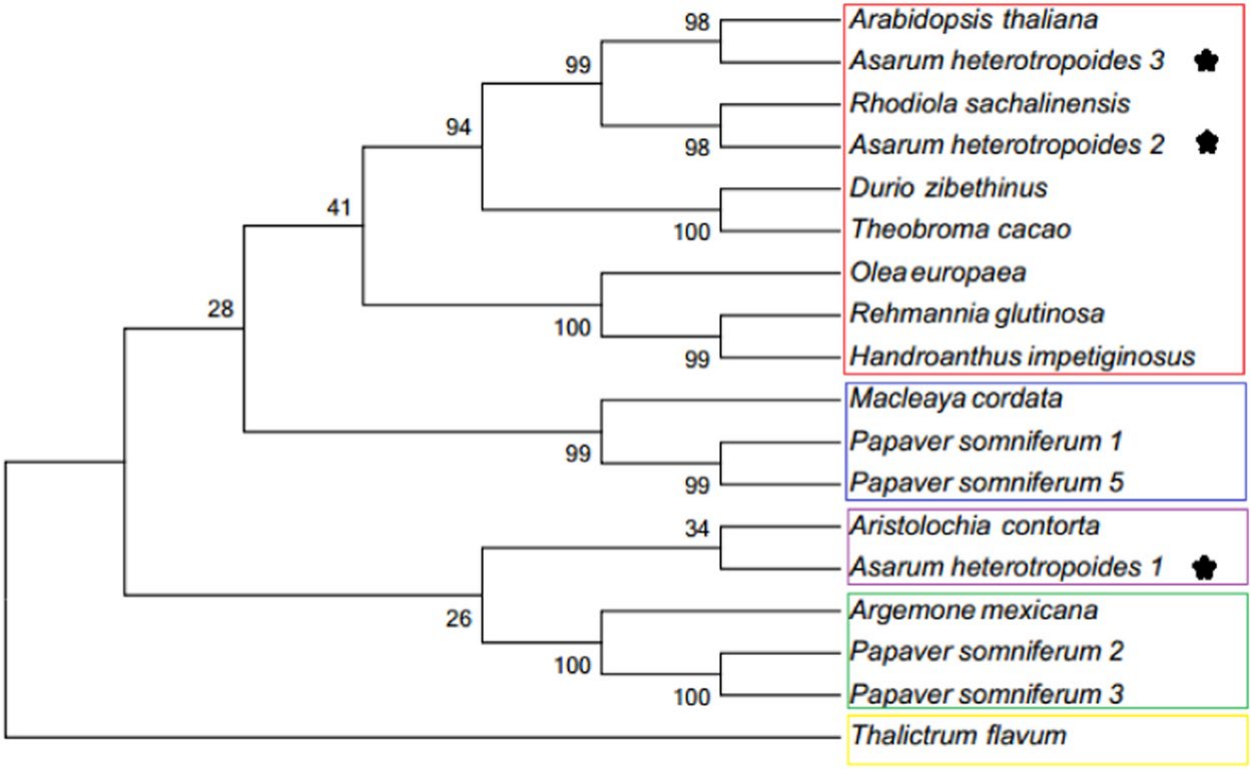
Phylogenetic tree of TyrDCs included *Asarum heterotropoides* TyrDCs (1, 2, 3) (in this article). The phylogenetic tree was constructed using MEGA software (version7.0) based on the Maximum Likelihood (ML) method. Values above the branches are bootstrap percentages (1000 replicates). Abbreviations and access codes are as follows: *Asarum heterotropoides* (1, 2, 3), *Asarum heterotropoides* Tyrosine decarboxylase 1, 2, 3 (in this article); *Aristolochia contorta* (ABJ16446.1), *Aristolochia contorta* tyrosine decarboxylase; *Papaver somniferum* 1 (P54768.1), *Papaver somniferum* Tyrosine/DOPA decarboxylase 1; *Papaver somniferum* 2 (P54769.1), *Papaver somniferum* Tyrosine/DOPA decarboxylase 2; *Papaver somniferum* 3 (P54770.2), *Papaver somniferum* Tyrosine/DOPA decarboxylase 3; *Papaver somniferum* 5(P54771.1), *Papaver somniferum* Tyrosine/DOPA decarboxylase 5; *Rhodiola sachalinensis* (ABF06560.1), *Rhodiola sachalinensis* Tyrosine/DOPA decarboxylase; *Macleaya cordat* (OUZ99264.1), *Macleaya cordata* Pyridoxal phosphate-dependent decarboxylase; *Thalictrum flavum* (AAG60665.1), *Thalictrum flavum* tyrosine/dopa decarboxylase; *Argemone mexicana* (ACJ76782.1), *Argemone mexicana* tyrosine/DOPA decarboxylase; *Olea europaea* (ALG62777.1), *Olea europaea* putative tyrosine decarboxylase; *Theobroma cacao* (EOX96928.1), *Theobroma cacao* Tyrosine/DOPA decarboxylase; *Handroanthus impetiginosus* (PIN03085.1), *Handroanthus impetiginosus* Aromatic-L-amino-acid/L-histidine decarboxylase; *Durio zibethinus* (ALG62777.1), *Durio zibethinus* tyrosine/DOPA decarboxylase 1-like; *Rehmannia glutinosa* (AOC38014.1), *Rehmannia glutinosa* tyrosine decarboxylase; *Arabidopsis thaliana* (AEE85525.1), *Arabidopsis thaliana* L-tyrosine decarboxylase; The *Asarum heterotropoides* TyrDC1, 2, and 3 as labeled after taxon marker.

**Figure 13:**
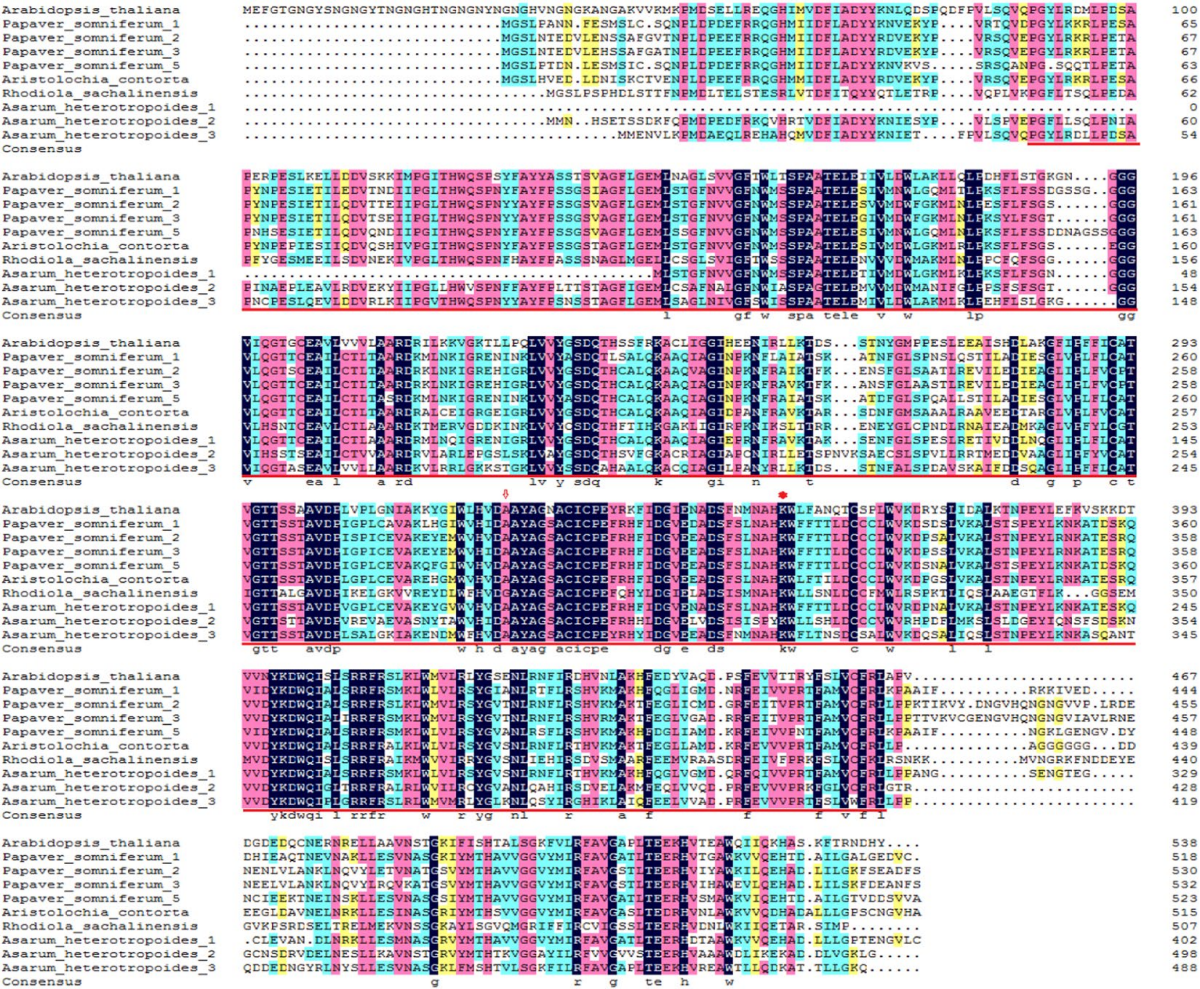
Multi-alignment of TyrDCs included *Asarum heterotropoides* TyrDCs (1, 2, 3) (in this article). The Pyridoxal-deC domain is underlined, the arrow shows the aspartate D residue that interacts with the activation site, and the asterisk shows a conserved activation site lysine K residue. Abbreviations and access codes are as follows: *Asarum heterotropoides* (1, 2, 3), *Asarum heterotropoides* Tyrosine decarboxylase1, 2, 3 (in this article); *Arabidopsis thaliana* (AEE85525.1) *Arabidopsis thaliana* L-tyrosine decarboxylase; *Aristolochia contorta* (ABJ16446.1), *Aristolochia contorta* tyrosine decarboxylase; *Rhodiola sachalinensis* (ABF06560.1), *Rhodiola sachalinensis* Tyrosine/DOPA decarboxylase; *Papaver somniferum* 1 (P54768.1), *Papaver somniferum* Tyrosine/DOPA decarboxylase 1; *Papaver somniferum* 2 (P54769.1), *Papaver somniferum* Tyrosine/DOPA decarboxylase 2; *Papaver somniferum* 3 (P54770.2), *Papaver somniferum* Tyrosine/DOPA decarboxylase 3; *Papaver somniferum* 5 (P54771.1), *Papaver somniferum* Tyrosine/DOPA decarboxylase 5; Single, fully conserved residues are indicated by an asterisk.

We cloned the full-length gene of the *TyrDC* family found in the plant metabolic network and obtained the complete coding sequences (CDS) of three genes based on predicted genes in the transcriptome data. This finding demonstrated the existence of the gene in *A. heterotropoides*. Furthermore, information on these three genes was submitted to GenBank (*TyrDC1* ID: MH119138, *TyrDC2* ID: MH119139, and *TyrDC3* ID: MH119140). The phylogenetic tree was based on *TyrDC* sequences of 13 species (Fig. 12). Phylogenetic trees are divided into five categories. TyrDC2 and TyrDC3 are highly homologous with the TyrDC of nine species. TyrDC2 has high homology with *Rhodiola sachalinensis*, which is cold-tolerant and rich in alkaloids. Similarly, *A. heterotropoides* also has such physiological characteristics. Therefore, TyrDC2 may be involved in the accumulation of alkaloids and plant stress tolerance. TyrDC1 has high homology with *Aristolochia contorta*. Both are the *Aristolochia* plants, which contain aristolochic acid. Therefore, TyrDC1 may be a relatively unique TyrDC in *Aristolochia* plants. Comparison of the amino acid sequences of TyrDC1, TyrDC2, and TyrDC3 revealed that their functional domains were identical to those of seven TyrDCs from four other plant species (Fig. 13).

## Conclusion

In this study, transcriptomic high-throughput sequencing of *A. heterotropoides* was performed for the first time. In-depth transcriptome sequence data allowed us to identify the expression levels of key enzymes involved in the aristolochic acid biosynthetic pathway. To gain insights into the biosynthesis of biologically active compounds in *A. heterotropoides*, expression levels of genes associated with bioactive compound content in different tissues were evaluated, which revealed the complexity of gene expression and metabolite accumulation in the biosynthetic pathway. These findings need to be confirmed through further molecular, biochemical, enzymological, and physiological studies to better understand the underlying regulatory mechanisms involved. Nevertheless, our transcriptome data represent the first genomics resource of *A. heterotropoides* and lay the foundation for further research into *A. heterotropoides* using genetics, genomics, and biotechnology methods.

## Materials and Methods

### Plant material

We collected 6-year-old *Asarum* [*A. heterotropoides* Fr. Schmidt var. *mandshuricum* (Maxim.) Kitag.] seedlings from the Jilin Agricultural University of China Medicinal Materials College at the on 3 September 2016. The growth conditions were as follows: in the early stage of cultivation, we built an awning shed and manually weeded around the plants regularly during the growing process, and the rest were untreated. The seedlings had underground roots and leaves, and the dark green leaves that were free from worms were stored at −80 °C.

### RNA isolation and transcriptome sequencing

Total RNA from the roots and leaves of three plants was extracted and triplicate samples were processed separately. The quality of the RNA samples was evaluated using a bioanalyzer (Agilent, USA). The mRNA was captured using the NEBNext Poly(A) mRNA Magnetic Isolation Module, and the cDNA libraries were constructed using the NEBNext Ultra RNA Library Prep kit for Illumina and purified using Beckman Agencourt AMPure XP beads, followed by detection and quantification using the Agilent bioanalyzer 2100 and Qubit. cBOT auto-clustering was performed using the TruSeq PE cluster kit v4. The clustered library was processed using the TruSeq SBS kit v4-HS and then sequenced using the Illumina HiSeq 2000 platform using the two-terminal sequencing method.

### *De novo* assembly and sequence processing

The bcl2fastq^16^ (v2.17.1.14) software and FastQC (v0.10.1) were used to analyse the initial sequence data quality to obtain the pass filter data. The raw data read from six RNA-sequences (RNA-seqs) were subjected to quality trimming using the second-generation sequencing data quality statistics software, Cutadapt^17^ (version 1.9.1), to sequence the pass filter data to remove the linker and low-quality sequences to obtain clean data for subsequent information analysis. With the UniGene sequence file as a reference gene file, RSEM^18^ (v1.2.6) estimated gene and isoform expression levels from the pair-end clean data. Paired-end sequencing used 2 × 150 bp sequenced arm. The Cutadapt procedure was as follows: the adaptor sequence and bases whose masses at the 5′ or 3′ end were lower than 20 were removed, the reads containing more than 10% of N were removed, and reads less than 75 bp long were removed after trim removal.

Because there is no report on the genome or transcriptome data of *A. heterotropoides*, it was necessary to splice the clean data. Trinity^19^ (v2.2.0) was used to assemble the sample data from scratch. The assembly results were further sequenced and de-redundantly processed using sequence clustering to obtain long non-redundant UniGene sequences.

Assembly was performed by Trinity, which represents a novel method for the efficient and robust de novo reconstruction of transcriptomes from RNA-seq data. Trinity combines three independent software modules: Inchworm, Chrysalis, and Butterfly, applied sequentially to process large volumes of RNA-seq reads. Duplicated contigs were removed by cd-hit to obtain the UniGene sequence file.

### Gene function annotation

Open reading frames were predicted using the TransDecoder, and the data were annotated using the Nr, PFAM, and Swiss-Prot databases using the BLASTX program. The KEGG automatic annotation server 63 was used to map the transcript pathways in the non-redundant UniGene collection.

### Differential gene expression analysis

In the absence of a reference genome, UniGene was used to concatenate the Trinity data, the clean and UniGene data were compared, and then Bowtie2^20^ (2.1.0) software with default parameters was used to compare short reads to quantify the genes.

Differential expression analysis used the DESeq2^21^ (v1.18.0) Bioconductor package, a model based on the negative binomial distribution. After adjustments using Benjamini and Hochberg’s approach for controlling the false discovery rate, P-values of the genes were set at <0.05 to detect differentially expressed genes (changes in differential gene expression >2-fold and FDR ≤0.05). The entire FPKM^22–26^ density distribution reflected the UniGene expression data of the roots and leaves of *A. heterotropoides*.

### qRT-PCR analysis

The qRT-PCR was performed using the ABI StepOne system and the Brilliant II SYBR Green qRT-PCR Master Mix kit One-Step. β-Actin was used as the reference gene, and the specific primer sequences are shown in Table 7. Fluorescence qRT-PCR was performed on each gene using three biological and technical replicates each. Relative gene expression levels were calculated using the 2^−ΔΔCT^ method^27–30^.

**Table 7:** Specific primer sequences.

Name	Primer
*TyrDC1* F-1	5′-GCTGGTCTGGGTAACACG-3′
*TyrDC1* R-1	5′-AAGAAAGAGCGGGATCAGC-3′
*TyrDC1* F-2	5′-AGTCGGAGTTGCGGGTGT-3′
*TyrDC1* R-2	5′-GCCAGTCTTTGTAATCCACCAC-3′
*TyrDC2* F-1	5′-GAAGGCTGTTCAAGGTCG-3′
*TyrDC2* R-1	5′-ATCTCGTAACACCGCCTC-3′
*TyrDC2* F-2	5′-ACCGCTCGTTGATATGGC-3′
*TyrDC2* R-2	5′-ACGCAGCAACAGTCCAGAT-3′
*TyrDC3* F-1	5′-CGAACATAGACGGGACTC-3′
*TyrDC3* R-1	5′-GCTTGTGATGCCTTATTT-3′
*TyrDC3* F-2	5′-TCGACGTGCTCTATTTCA-3′
*TyrDC3* R-2	5′-CAGGAGATAGGGCAAAGT-3′
β-Actin -F	5′-AGCAGCTTCCATTCCGATCA-3′
β-Actin -R	5′-GGTTACATGTTCACCACCAC-3′

### Extraction and estimation of aristolochic acid I

The roots and leaves of the *A. heterotropoides* plant material were pulverised with liquid nitrogen, and 0.5 g of the powdered mixture was placed in a labelled 10 mL centrifuge tube, which was vortexed with 5 mL fresh 70% methanol (chromatography grade) for 30 s, kept aside for 1 h, and then weighed. After ultrasonication for 1 h, the tube was removed and then weighed again after cooling. The weight loss was compensated by adding 70% methanol, and 1.5 mL of the extract was centrifuged at 12,000 rpm for 1 min. The supernatant was filtered through a 0.22 μm organic membrane into a vial and stored airtight at 4 °C in the refrigerator.

The fresh leaves and roots were placed in a 40 °C oven and dried to a constant weight. The extraction was performed using the extraction step described above.

High-performance liquid chromatography (HPLC) detection was performed using a Shimadzu LC-10ATvp system with a ZORBAX SB-C18 column (250 × 4.6 mm, 5 μm; Agilent Technologies). Chromatographic conditions for determining aristolochic acid I (Cas:313-67-7, purity ≥98%, Shanghaishifeng, China) content were as follows: mobile phase, methanol–0.1% aqueous acetic acid (72:28); column temperature, 27 °C; flow rate, 1.0 mL/min; detection wavelength, 315 nm; and injection volume, 20 μL. The retention time was determined using standard samples, and the aristolochic acid I content of each tissue sample was determined using a linear regression equation. Each sample was analysed in triplicate^31,32^.

### Cloning of *TyrDC* genes and identification of gene sequences

SnapGene Viewer (http://www.snapgene.com/) was used to detect the cleavage sites and positions of *TyrDC1*, *TyrDC2*, and *TyrDC3* gene sequences. Primer 5 (Premier Biosoft International, Palo Alto, CA, USA) was used to determine the CG content and Tm value of the primers, according to the predicted gene screening of restriction sites and the principles of primer design. An OMEGA gel extraction kit (Norcross, GA, USA) was used to recover the PCR products from the gel, which were then examined using the NanoDrop 2000 instrument (Thermo Fisher Scientific)^33^.

*TyrDC1*, *TyrDC2*, and *TyrDC3* were ligated using the pMD18-T vector and then transformed into Trans5α competent *Escherichia coli* cells for identification and sequencing^33^.

The obtained *TyrDC* amino acid sequences were compared with the amino acid sequences of *TyrDC* of other species using MUSCLE^34,35^ (http://www.drive5.com/muscle/) to generate the FASTA format, and the data were analysed using DNAman8 (https://www.lynnon.com/index.html). Phylogenetic trees (ML) were constructed using MEGA7 (https://www.megasoftware.net/)^36,37^.

### Accession Codes

Te datasets of raw read sequences from each tissue were deposited in the NCBI Short Read Archive (SRA, SRA accession: SRP151557) database under the BioProject accession number PRJNA477885.

## Electronic supplementary material

Supplementary Information

Dataset 1
