# Supplementary material for: Deep sequencing and transcriptome analysis to identify genes related to biosynthesis of aristolochic acid in Asarum heterotropoides
Source: Sci Rep. 2018 Dec 14;8:17850. doi: 10.1038/s41598-018-36316-0 (PMC6294785; doi:10.1038/s41598-018-36316-0)
Supplement: Supplementary file 1 — Supplementary Information [file 41598_2018_36316_MOESM1_ESM.pdf]

Deep sequencing and transcriptome analysis to identify genes related to biosynthesis of aristolochic acid in *Asarum heterotropoides*

Wang Xiaohan*, Hui Fang, Yang Yongcheng, and Yang Shihai*

Traditional Chinese Medicine College, Jilin Agricultural University, Changchun City, Jilin Province 130118, China

*Corresponding authors:

Yang Shihai Email address: jlyangs@163.com

Wang Xiaohan Email address: xiaohanw@163.com

Supplemental Table 1. Candidate UniGenes list.

No.	UniGene ID	foldChange	pval	NR_protein_ID
1	c290059_g1_i1			gi|720016627|ref|XP_010261209.1
2	c215679_g1_i1	0	8.02E-12	gi|545354089|ref|XP_005642879.1
3	c211451_g1_i1	0.276567956	6.32E-07	gi|695034052|ref|XP_009404519.1
4	c199520_g1_i1	0.379872428	8.01E-06	gi|720091809|ref|XP_010245533.1
5	c211451_g1_i2	2.704506778	0.0000089	gi|720094733|ref|XP_010246457.1
6	c215800_g2_i1	2.191671051	0.0001582	gi|719982170|ref|XP_010250350.1
7	c149320_g1_i1	Inf	0.0003981	gi|747063524|ref|XP_011078312.1
8	c146272_g1_i2	Inf	0.0006614	gi|747042923|ref|XP_011081001.1
9	c377160_g1_i1	Inf	0.0010237	gi|747040616|ref|XP_011090162.1
10	c191631_g2_i1	0	0.0021403	gi|662502970|gb|KEQ60593.1
11	c174134_g2_i1	Inf	0.003718	gi|224143258|ref|XP_002324896.1
12	c332386_g1_i1			gi|586549432|ref|XP_006909787.1
13	c212240_g1_i1	0	4.98E-73	gi|672119786|ref|XP_008783141.1
14	c212240_g1_i2	0.006724043	3.81E-39	gi|672119786|ref|XP_008783141.1
15	c213637_g1_i1	20.12522497	2.04E-36	gi|930809514|gb|ALG05139.1
16	c213648_g2_i1	0.053638064	6.78E-25	gi|116247551|gb|ABJ90144.1
17	c196437_g2_i1	Inf	7.24E-22	gi|1024042478|gb|KZV45880.1
18	c211478_g1_i3	13.434884	1.47E-17	gi|930809514|gb|ALG05139.1
19	c196437_g2_i2	Inf	1.25E-06	gi|1024042478|gb|KZV45880.1
20	c213312_g1_i1			gi|720091108|ref|XP_010245321.1
21	c217593_g1_i1	267.58737	6.05E-21	gi|720070894|ref|XP_010277895.1
22	c50470_g1_i1	0.009567	5.27E-06	gi|720070894|ref|XP_010277895.1
23	c42003_g1_i1	Inf	1.45E-07	gi|936413435|gb|KPM41887.1
24	c213390_g1_i3	167.73644	3.14E-30	gi|802650549|ref|XP_012079991.1
25	c202205_g1_i1	190.33893	2.96E-16	gi|747068716|ref|XP_011081126.1
26	c185071_g1_i1	Inf	0.0000227	gi|747106016|ref|XP_011101289.1
27	c198638_g1_i1	2.0703861	0.0004164	gi|359495915|ref|XP_002273532.2
28	c99956_g1_i1	Inf	0.0015673	gi|747106016|ref|XP_011101289.1
29	c167169_g2_i1			gi|629122181|gb|KCW86671.1
30	c213390_g1_i6			gi|720064068|ref|XP_010275835.1
31	c159791_g1_i1	41.383647	1.20E-32	gi|747097360|ref|XP_011096642.1
32	c192979_g1_i1			gi|743777069|ref|XP_010918954.1
33	c217593_g1_i1	267.58737	6.05E-21	gi|720070894|ref|XP_010277895.1
34	c218419_g2_i3	14.916523	2.93E-23	gi|720090665|ref|XP_010245171.1

Supplemental Table 2. FPKM and the counts of candidate UniGenes.

No.	FPKM						counts					
	leaf-1	leaf-2	leaf-3	root-1	root-2	root-3	leaf-1	leaf-2	leaf-3	root-1	root-2	root-3
1	16.11	20.61	21.6	82.05	84.25	83.47	356	467	461	2208	1821	1959
2	2.37	5.07	2.9	0	0	0	50	110	59.25	0	0	0
3	20.04	14.81	23.59	1.73	5.85	8.18	206.2	156.3	235.8	21.39	57.74	87.65
4	34.79	40.32	38.34	12.86	13.23	15	848	1008	902	382	316	389
5	8.61	10.81	6.09	23	21.8	21.13	215.8	277.7	147.2	702.6	535.3	563.4
6	52.83	58.49	64.46	120.1	125.1	121.7	1300	1476	1531	3602	3016	3185
7	0	0	0	0.95	1.4	0.74	0	0	0	12	14	8
8	0	0	0	0.75	0.79	0.53	0	0	0	13	11	8
9	0	0	0	1.22	1.54	1.04	0	0	0	11	11	8
10	0.43	1.68	0.67	0	0	0	4	16	6	0	0	0
11	0	0	0	0.29	1.18	0.59	0	0	0	4	13	7
12	0.17	0.27	0.12	0.14	0.4	0.11	3	5	2	3	7	2
13	37.47	37.55	41.12	0	0	0	872.7	896.9	924.5	0	0	0
14	23.32	26.19	16.96	0.07	0.18	0.17	516.9	595.3	362.9	2	4	4
15	4.97	6.33	2.75	92.97	92.79	83.8	163	213	87	3725	2993	2936
16	94.52	103.1	67.42	4.89	4.99	3.82	1387	1552	958	87	71	59
17	0	0	0	4.94	5.51	4.04	0	0	0	104	93	74
18	1.22	0.73	0.54	12.11	10.04	9.98	21	13	9	253.9	168.7	182.1
19	0	0	0	1.29	0.68	1.37	0	0	0	26	11	24
20	20.44	34.22	15.76	20.83	19.14	18.22	639	1097	475	795	588	608
21	0.04	0	0	2.7	3.99	4.03	1	0	0	79	94	103
22	16.05	17.7	50.01	0.11	0.25	0.39	458	518	1377	4	7	12
23	0	0	0	0.96	0.77	0.99	0	0	0	28	18	25
24	0.08	0	0	4.77	4.68	3.68	2.92	0	0	214.1	169.3	144.5
25	0.03	0	0	2.05	1.87	1.31	1	0	0	86.98	64	48.54
26	0	0	0	1.1	1.15	0.99	0	0	0	18	15	14
27	47.97	48.46	53.93	96.33	101	97.03	2344	2428	2537	5760	4871	5082
28	0	0	0	0.31	0.52	0.39	0	0	0	8	11	9
29	6.61	6.46	4.54	3.6	0.81	2.28	10	10	7	6	1	3
30	9.08	9.07	11.06	6.99	7.94	8.65	273.2	279.8	320.8	256.6	234.6	277.4
31	0	0	0	7.63	6.38	5.04	0	0	0	222	149	128
32	6.47	6.39	4.6	6.53	6.48	6.59	156	158	107	192	153	169
33	0.04	0	0	2.7	3.99	4.03	1	0	0	79	94	103
34	2.02	1.96	0.12	18.9	21.21	18.81	38.15	37.82	2.11	432.9	389.8	375.3

Supplemental Table 3. Relative expression of *TyrDCs*

	Leaf	Root
TyrDC1	5.225±0.445	2.579±0.499
TyrDC2	5.661±0.377	2.838±0.319
TyrDC3	5.083±0.584	2.537±0.531

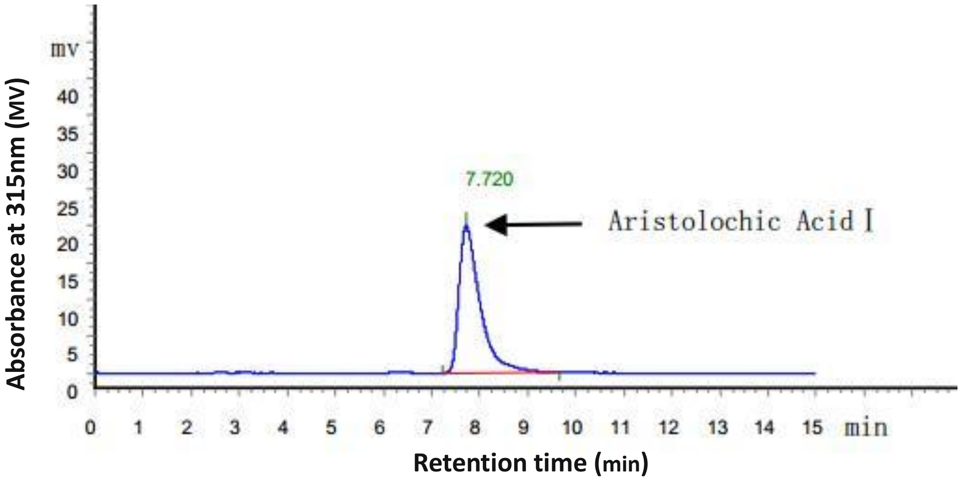

Supplemental Figure 1. HPLC determination of Aristolochic acid Ⅰ standard. The marker “←” indicates Aristolochic acid Ⅰ with a retention time of about 7.720 min.
